# Cell-type-specific synaptic imbalance and disrupted homeostatic plasticity in cortical circuits of ASD-associated *Chd8* haploinsufficient mice

**DOI:** 10.1038/s41380-021-01070-9

**Published:** 2021-04-09

**Authors:** Robert A. Ellingford, Martyna J. Panasiuk, Emilie Rabesahala de Meritens, Raghav Shaunak, Liam Naybour, Lorcan Browne, M. Albert Basson, Laura C. Andreae

**Affiliations:** 1 Centre for Developmental Neurobiology, Institute of Psychiatry, Psychology & Neuroscience, King’s College London, London, UK; 2 Centre for Craniofacial & Regenerative Biology, King’s College London, London, UK; 3 MRC Centre for Neurodevelopmental Disorders, King’s College London, London, UK

**Keywords:** Autism spectrum disorders, Neuroscience

## Abstract

Heterozygous mutation of chromodomain helicase DNA binding protein 8 (*CHD8*) is strongly associated with autism spectrum disorder (ASD) and results in dysregulated expression of neurodevelopmental and synaptic genes during brain development. To reveal how these changes affect ASD-associated cortical circuits, we studied synaptic transmission in the prefrontal cortex of a haploinsufficient *Chd8* mouse model. We report profound alterations to both excitatory and inhibitory synaptic transmission onto deep layer projection neurons, resulting in a reduced excitatory:inhibitory balance, which were found to vary dynamically across neurodevelopment and result from distinct effects of reduced *Chd8* expression within individual neuronal subtypes. These changes were associated with disrupted regulation of homeostatic plasticity mechanisms operating via spontaneous neurotransmission. These findings therefore directly implicate *CHD8* mutation in the disruption of ASD-relevant circuits in the cortex.

## Introduction

Autism spectrum disorder (ASD) is a common neurodevelopmental disorder characterized by social communication deficits and repetitive behaviors. Imbalances in the levels of excitatory and inhibitory activity (E:I balance) within the neuronal circuitry of the cerebral cortex are a proposed causative mechanism of ASD [1–3]. E:I balance governs the output of neurons based on the local level of excitatory/inhibitory synaptic transmission and intrinsic excitability. Some of the highest confidence ASD-risk genes encode proteins that directly impact E:I balance [4, 5] and multiple animal models display synaptic transmission deficits alongside ASD-like behavioral abnormalities [2, 6–12].

E:I balance is sustained through homeostatic plasticity mechanisms that maintain network activity within an optimal range by tuning synaptic strength [13] and intrinsic excitability [14]. However, the role that homeostatic plasticity plays in the context of ASD-risk mutations remains unclear. It has been proposed that homeostatic mechanisms could be insufficient or maladaptive [2]. Reductions in normal homeostatic responses have been demonstrated in dissociated neuronal cultures from mice with mutations in the ASD-associated genes *MeCP2* [15–17], *Fmr1* [18, 19], and *Shank3* [20] and in visual cortex following eyelid suture [20], implicating the failure of such mechanisms in ASD etiology. Conversely, it has been suggested that many changes to E:I balance seen in ASD gene mutant mice may themselves be compensatory and reflect the normal operation of homeostatic plasticity [21]. Notably, there has been a lack of study in regions of the brain directly relevant to ASD, such as prefrontal cortex (PFC). Furthermore, to what extent changes to synaptic E:I balance are specific to the cell type affected by the gene deletion versus secondary or even compensatory effects remains poorly understood.

De novo heterozygous mutation of chromodomain helicase DNA binding protein 8 (*CHD8*) is one of the highest confidence genetic risk factors for ASD [22–26]. *CHD8* encodes a chromatin remodeler that regulates the expression of many other ASD-associated genes essential for brain and synapse development in both human [27–30] and mouse models [7, 8, 31–33]. However, despite these studies, the impact of reduced *CHD8* expression on the development and function of specific circuits that could give rise to ASD remains unclear. We therefore conducted a detailed characterization of E:I balance during postnatal cortical development in a *Chd8* heterozygous (*Chd8*^*+/–*^) mouse model [32]. We focused our analysis on deep layer (V/VI) pyramidal neurons within the PFC as this cell type and brain region have been strongly implicated in ASD etiology through both anatomical [34] and transcriptomic [35] studies. These mice were found to display developmental stage-specific alterations to both excitatory and inhibitory synaptic transmission that affected neuronal output. *Chd8* haploinsufficiency targeted to excitatory or inhibitory neurons revealed both cell-type-specific synaptic alterations and indirect changes. Finally, we found that homeostatic responses, dependent on spontaneous neurotransmission, were defective in the *Chd8*^*+/*^^–^ PFC.

## Materials and methods

### Mice

All procedures were performed according to the Animals (Scientific Procedures) Act 1986 with ethical approval granted by the UK Home Office. The conditional *Chd8* (*Chd8*^*flox*^) and *Chd8* null (*Chd8*^–^) alleles were generated by our lab and have been described in detail previously [32]. *Chd8*^*flox*^ mice were bred with a variety of *Cre* mice to excise exon 3, resulting in an early frameshift and termination of translation at amino acid 419 (*Chd8*^*–*^) to produce a protein lacking all functional domains, equivalent to nonsense and frameshift mutations identified in patients [36]. Constitutive *Chd8* heterozygotes (*Chd8*^*+/–*^) have previously been verified to express approximately 50% of the wild-type (WT) levels of *Chd8* at both the RNA and protein level [32]. All experiments were performed blind to genotype.

For dendritic spine and inhibitory synapse analysis, *Chd8*^*+/–*^ mice were bred with Tg(Thy1-EGFP)MJrs/J (*Thy1-GFP-M*) mice [37]. For experiments utilizing conditional *Chd8*^*+/–*^ mice (*cChd8*^*+/–*^), homozygous *Chd8*^*flox/flox*^ mice were bred with mice heterozygous for the respective Cre line (*Nkx2.1-Cre* [38] *or NEX-Cre* [39]). All genotyping used the HotSHOT method for DNA extraction [40].

### Electrophysiology

Electrophysiological recordings were performed on acute brain slices using whole-cell patch clamp electrophysiology. For details of standard electrophysiological methods, slice preparation (including use of Na^+^ reintroduction for adult slices), miniature postsynaptic current, intrinsic properties, and paired pulse ratio recordings, please see Supplementary Materials and methods.

### Spontaneous spiking recordings

All spontaneous spiking recordings were performed in standard artificial cerebrospinal fluid (ACSF, see Supplementary Methods) using K-gluconate internal solution. Whole-cell conformation was achieved in voltage-clamp mode with −70 mV holding potential, after which membrane potential was increased to −40 mV and recording mode immediately switched to current clamp, resulting in a constant injection of current that maintained membrane potential just below AP firing threshold. Spontaneous spiking was then recorded for 1 min. Two recordings of spontaneous spiking were taken from each cell, with a 1-min recovery period between each recording where membrane potential was held at −70 mV in voltage-clamp mode.

### Homeostatic plasticity recordings

P13–15 mouse brains were sliced as detailed in Supplementary Materials and methods. Before being placed in ACSF to recover, slices were cut down the midline in order to separate the two hemispheres. One hemisphere was placed in standard ACSF and the other placed in ACSF containing drug treatment, alone or in combination: 1 µM tetrodotoxin (TTX); 25 µM (2*R*)-amino-5-phosphonovaleric acid (APV); 10 µM SR-95531 (Gabazine); 45 µM anisomycin. Slices were incubated for 6 h at room temperature with perfusion of 95% O_2_/5% CO_2_ before drug-treated slices were transferred to standard ACSF. Miniature postsynaptic currents were then recorded from the treated and untreated slices as before. To allow comparison between animals, the frequency and amplitude of currents for each animal were normalized to the mean untreated value.

### Sholl analysis

For details of dendritic imaging and Sholl analysis, please see Supplementary Materials and methods.

### Synapse analysis

For details of immunohistochemistry, image acquisition and analysis, please see Supplementary Materials and methods.

### Statistics

Data are presented as mean ± standard error of the mean unless otherwise stated. All experiments were analyzed blind to genotype and treatment. All statistical analyses were performed using Prism software (GraphPad). For pairwise comparisons the D’Agostino–Pearson omnibus normality test was first used to determine Gaussian distribution within data sets. If both data sets were normally distributed, comparisons were performed using unpaired *t* tests (with Welch’s correction if standard deviations were found to significantly differ). If data were not normally distributed, then pairwise comparisons were performed using the nonparametric Mann–Whitney *U* test (Supplementary Table 1). Multivariate comparisons were performed using a two-way ANOVA followed by Tukey’s multiple comparisons test (Supplementary Table 2).

## Results

### Reduced synaptic E:I balance in the PFC of *Chd8*^*+/–*^ mice

**Fig. 1:**
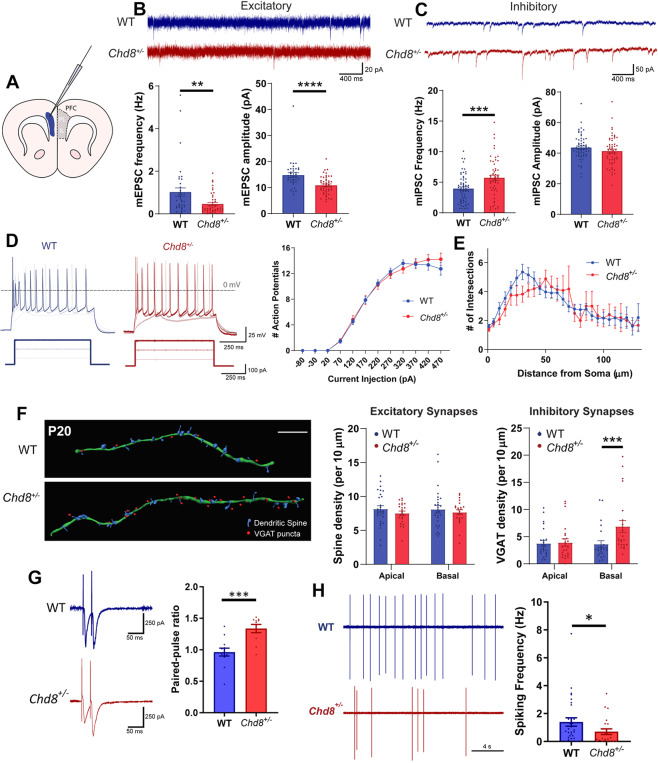
Reduced synaptic E:I balance in the PFC of *Chd8*^*+/–*^ mice. **A** Schematic illustrating coronal ex vivo brain slice, blue shading indicates recording area in layers V/VI of the PFC. IL infralimbic cortex, PL prelimbic cortex. **B**, **C** Representative mEPSC (**B**) and mIPSC (**C**) recordings from pyramidal neurons from P19–21 WT (blue) and *Chd8*^*+/–*^ (red) animals alongside quantifications of mEPSC and mIPSC frequency (bottom left graphs) and amplitude (bottom right graphs). **B** *Chd8*^*+/–*^ neurons display significantly reduced mEPSC frequency (*p* = 0.0027) and amplitude (*p* < 0.0001) but **C** increased mIPSC frequency (*p* = 0.0009) and equivalent mIPSC amplitude. **D** Normal action potential (AP) firing frequency in *Chd8*^*+/–*^ neurons; left: representative voltage traces and current injection stimuli (below), right: *f-I* curves. **E** Sholl analysis of basal dendrites (P22) shows no significant difference in *Chd8*^*+/–*^ neurons. **F** Analysis of synapse densities at P20; left: representative reconstructions of secondary basal dendrites (green filament) with associated spines (blue) and inhibitory synapses (red dots) from WT (above) and *Chd8*^*+/–*^ (below) *GFP*+ pyramidal neurons immunolabeled for the inhibitory synapse marker VGAT (scale bar 10 µm), with quantifications of spine (middle) and VGAT (right) densities. *Chd8*^*+/–*^ neurons showed no difference in spine densities, nor in VGAT density on apical dendrites, but did show an increase in VGAT density on basal dendrites (*p* = 0.0002). **G** Representative PPR recordings (left) from WT and *Chd8*^*+/–*^ PFC neurons; quantification (right) shows elevated PPR in *Chd8*^*+/–*^ neurons. **H** Reduced spontaneous AP firing in *Chd8*^*+/–*^ neurons, representative traces (left) and quantification (right). For all figures, bars show mean, error bars SEM, *p < 0.05, **p < 0.01, ***p < 0.001, ****p < 0.0001.

We first determined the effect of *Chd8* haploinsufficiency on the balance of synaptic transmission within the PFC. Whole-cell voltage-clamp recordings of miniature excitatory and inhibitory postsynaptic currents (mEPSCs and mIPSCs, respectively) were performed in deep layer neurons of the PFC, targeting the prelimbic cortex and infralimbic cortex (Fig. 1A) using ex vivo brain slices prepared from postnatal day 20 (P20) *Chd8*^*+/–*^ mice and their WT littermates. *Chd8*^*+/–*^ neurons displayed significantly decreased frequency and amplitude of mEPSCs (Fig. 1B) and increased mIPSC frequency (Fig. 1C), indicating decreased levels of excitatory synaptic transmission with a concurrent increase in inhibitory transmission on to these neurons. We then determined the intrinsic excitability of these neurons by measuring the frequency of action potential (AP) firing in response to increasing current injection (*f-I* curves) at P19-P21 using whole-cell current clamp recordings. The *f-I* curves of WT and *Chd8*^*+/–*^ neurons did not differ significantly (Fig. 1D). In addition, the magnitude and width of representative APs were equivalent between genotypes, as well as the voltage threshold and rheobase required to elicit responses (Supplementary Fig. 1). We also compared the passive membrane properties of WT and *Chd8*^*+/–*^ neurons, finding no differences in resting membrane potential, membrane capacitance, or membrane resistance (Supplementary Fig. 1). Therefore, reduced *Chd8* expression does not affect neuronal intrinsic excitability, size, or ion channel composition. To determine whether the synaptic changes might reflect differential sampling of cell projection types, we carried out a principal component analysis with hierarchical clustering of electrophysiological properties, previously shown to reflect projection patterns [41], for WT and *Chd8*^*+/–*^ neurons. This demonstrated no distinction between WT and *Chd8*^*+/–*^ neurons, nor any clear division into previously defined subgroups (Supplementary Fig. 2 and Supplementary Tables 3–6), indicating this was unlikely to be the case.

To address whether these synaptic alterations reflected large-scale changes to neuronal structure we carried out Sholl analysis in P22 Golgi-Cox stained *Chd8*^*+/–*^ neurons. This revealed there to be no differences in the degree of dendritic arborization (Fig. 1E). To determine whether the alterations in mEPSC and mIPSC frequency resulted from structural changes to synapse number, we assessed the density of dendritic spines and vesicular GABA transporter (VGAT)-positive synapses on the secondary apical and basal dendrites of these neurons at P20. We saw no change in dendritic spine density but did see an increased density of VGAT^+^ synapses on basal dendrites (Fig. 1F). Changes to mEPSC frequency can also be due to changes in synaptic release probability. Paired pulse ratio experiments in these neurons demonstrated a significant increase in the amplitude of the second pulse over the first (Fig. 1G), indicating a reduction in release probability at these excitatory inputs. Together these results suggest that reduced *Chd8* expression specifically impacts synaptic E:I balance on to deep layer PFC neurons, with reduced excitatory input due to decreased presynaptic release probability and postsynaptic changes, and increased inhibitory input at least in part due to an increase in the number of inhibitory synapses onto basal dendrites. Coupled with unaltered intrinsic excitability, the observed changes to synaptic transmission in *Chd8*^*+/–*^ neurons should result in reduced E:I balance within the PFC compared to WT littermates. Direct examination of spontaneous AP firing in these neurons indeed showed significantly decreased firing in the *Chd8*^*+/–*^ mutant (Fig. 1H), suggesting that the synaptic alterations impact neuronal output.

### Developmental stage-specific synaptic effects of *Chd8* heterozygosity

**Fig. 2:**
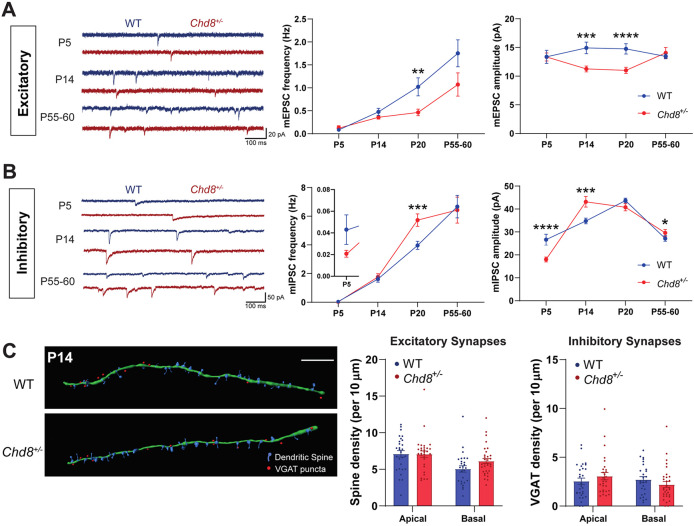
Developmental stage-specific synaptic effects of *Chd8* heterozygosity. **A**, **B** Developmental trajectory of excitatory (**A**) and inhibitory (**B**) synaptic function; left: representative mEPSC (**A**) and mIPSC (**B**) recordings from PFC neurons in ex vivo slices from P5, P14, and P55–60 WT (blue) and *Chd8*^*+/–*^ (*red*) animals: frequency (middle) and amplitude (right) changes during development. *Chd8*^*+/–*^ neurons showed no significant difference in mEPSC frequency at P5, P14, and P55–60 nor in amplitude at P5 and P55–60 but did show decreased mEPSC amplitude at P14. *Chd8*^*+/–*^ neurons showed no significant difference in mIPSC frequency at P5, P14, and P55–60, but displayed decreased mIPSC amplitude at P5 (*p* < 0.0001) followed by increased amplitude at P14 (*p* = 0.0008) and P55–60 (*p* = 0.033). **C** Analysis of synapse densities at P14; left: representative reconstructions of secondary basal dendrites (green filament) with associated spines (blue) and inhibitory synapses (red dots) from WT (above) and *Chd8*^*+/–*^ (below) *GFP*+ pyramidal neurons immunolabeled for the inhibitory synapse marker VGAT (scale bar 10 µm), with quantifications of spine (middle) and VGAT (right) densities. At P14, *Chd8*^*+/–*^ neurons showed no difference in either spine or inhibitory synapse densities. *p < 0.05, **p < 0.01, ***p < 0.001, ****p < 0.0001.

*Chd8* expression levels and the effects of *Chd8* haploinsufficiency on gene expression vary substantially over the course of neurodevelopment [31, 32]. Therefore, in addition to P20 animals, we characterized excitatory and inhibitory synaptic integrity within *Chd8*^*+/–*^ mice over a time course consisting of neonatal (P5), adolescent (P14), and adult (P55–60) ages. We found no significant differences in mEPSC frequency until the decrease at the P20 timepoint that appeared to persist into adulthood (Fig. 2A). We also observed that mEPSC amplitude was already decreased at P14 but had recovered by adulthood (Fig. 2A). We saw no alterations to mIPSC frequency apart from at P20 but did observe substantial alterations to mIPSC amplitude, which was decreased at P5 and increased at P14 and adult stages (Fig. 2B). The absence of any alterations to mEPSC or mIPSC frequency at P14 was supported by our findings of no difference in dendritic spine or VGAT^+^ synaptic density on apical or basal dendrites at this stage (Fig. 2C). This therefore suggests that while there may be small changes to inhibitory inputs as early as P5, the most significant alterations to synaptic E:I balance appear in a window around P20.

### Differential alterations to synaptic transmission from cell-type-specific *Chd8* heterozygosity

**Fig. 3:**
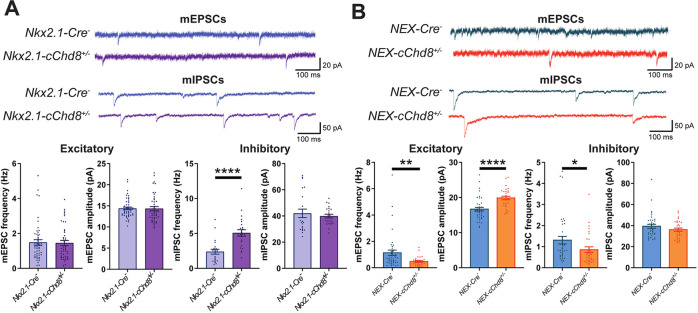
Differential alterations to synaptic transmission from cell-type-specific *Chd8* heterozygosity. **A**, **B** Representative mEPSC (top) and mIPSC (bottom) recordings from P19–21 *Nkx2.1-cChd8*^*+/–*^ (**A**, purple) and *NEX-cChd8*^*+/–*^ (**B**, orange) neurons and respective controls (blue and teal traces) alongside quantifications of mEPSC and mIPSC frequency and amplitude. *Nkx2.1-cChd8*^*+/–*^ neurons showed no difference in mEPSC frequency or amplitude but showed increased mIPSC frequency (*p* < 0.0001) with no change in amplitude. NEX-cChd8^+/–^ neurons showed decreased mEPSC frequency (*p* = 0.0027) and increased mEPSC amplitude (*p* < 0.0001) with decreased mIPSC frequency (*p* = 0.011) and no change in mIPSC amplitude. *p < 0.05, **p < 0.01, ****p < 0.0001.

To determine whether these contrasting synaptic phenotypes arise through *Chd8* deficiency in specific neuronal cell types, we selectively targeted either excitatory neurons, by forebrain-specific, postmitotic Cre-mediated deletion of a conditional *Chd8*^*flox*^ allele (*NEX-cChd8*^*+/–*^), or the majority of inhibitory neurons using an *Nkx2.1-Cre* line (*Nkx2.1-cChd8*^*+/–*^) and performed mEPSC and mIPSC recordings in the PFC as before, at P19–21. Haploinsufficiency of *Chd8* solely in interneurons (*Nkx2.1-cChd8*^*+/–*^) replicated the increase in mIPSC frequency seen in constitutive *Chd8* heterozygotes, while no changes to excitatory transmission were observed (Fig. 3A), indicating that the inhibitory transmission phenotype is driven cell-autonomously by interneurons. Equally, *Chd8* haploinsufficiency restricted to excitatory neurons (*NEX-cChd8*^*+/–*^) replicated the decreased mEPSC frequency seen in full *Chd8* heterozygotes (Fig. 3B), suggesting that *Chd8* regulates excitatory transmission cell-autonomously in post-mitotic excitatory neurons. However, in contrast to the constitutive *Chd8* heterozygotes, *NEX-cChd8*^*+/–*^ PFC neurons also displayed increased mEPSC amplitudes and decreased mIPSC frequency (Fig. 3B), suggestive of activation of compensatory mechanisms. While these are likely to be complex, the absence of such compensation in the constitutive *Chd8*^*+/–*^ mice led us to hypothesize that homeostatic mechanisms might be impaired in these animals.

### Disrupted homeostatic regulation of synaptic transmission in *Chd8*^*+/–*^ neurons

**Fig. 4:**
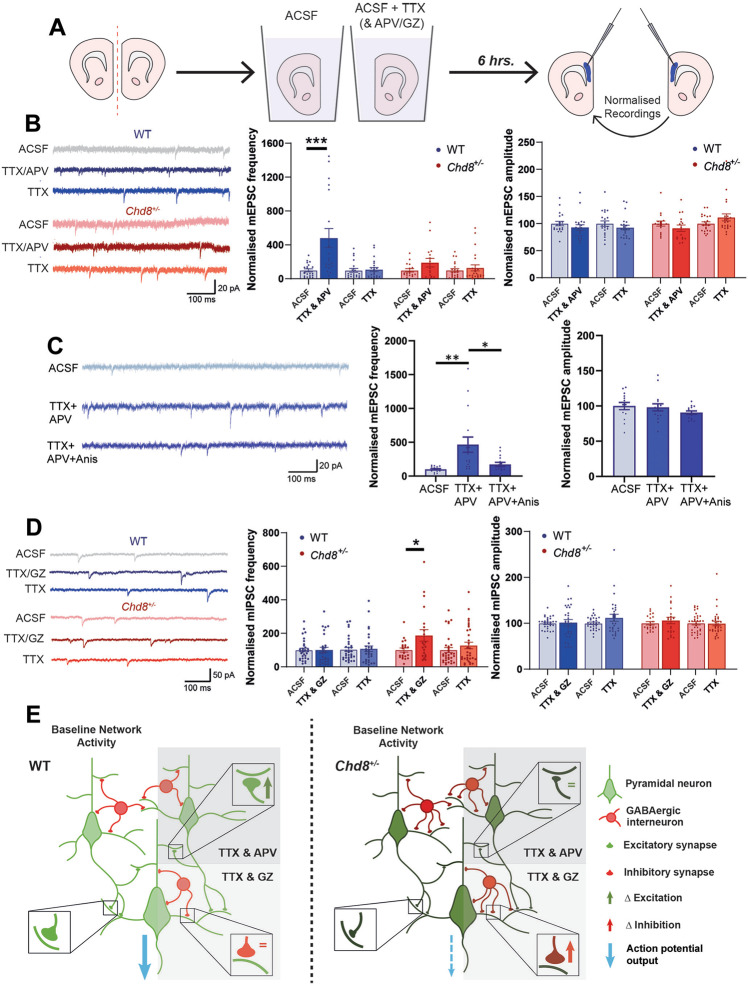
Disrupted homeostatic regulation of synaptic transmission in *Chd8*^*+/–*^ neurons. **A** Schematic of homeostatic plasticity induction paradigm in ex vivo P13–15 slices. **B** mEPSC responses to blocking AP firing ± NMDA receptors; left: representative mEPSC recordings from WT (blue) and *Chd8*^*+/–*^ (red) neurons incubated for 6 h in ACSF (top), ACSF+TTX+APV (middle) or ACSF+TTX (bottom) alongside quantifications of frequency (middle) and amplitude (right). WT neurons displayed a significant increase in mEPSC frequency following TTX/APV treatment (*p* = 0.0003; genotype effect, *p* = 0.029) while *Chd8*^*+/–*^ neurons failed to respond; no effects were seen on mEPSC amplitude. Treatment with TTX alone did not induce any change in mEPSC frequency or amplitude in either WT or *Chd8*^*+/–*^ neurons. **C** Homeostatic increase in mEPSC frequency following TTX/APV treatment is prevented by application of the protein translation blocker anisomycin; layout as for (**B**). **D** mIPSC responses to blocking AP firing ± GABA_A_ receptors; left: representative mIPSC recordings arranged as in (**B**). WT neurons displayed no change in mIPSC frequency following TTX/GZ treatment while *Chd8*^*+/–*^ neurons showed a significant increase (*p* = 0.021), with no effects on mIPSC amplitude. Treatment with TTX alone did not affect mIPSC frequency or amplitude in either WT or *Chd8*^*+/–*^ neurons. **E** Summary diagram depicting the synaptic phenotype resulting from *Chd8* haploinsufficiency. Reduced *Chd8* expression throughout development establishes a reduced E:I balance through decreased functional excitatory transmission (symbolized by smaller excitatory synapses) and increased numbers of inhibitory synapses onto basal dendrites, overall resulting in diminished neuronal output (dashed blue arrow). Altered homeostatic responses are seen in *Chd8*^*+/–*^ neurons (shaded areas on right). *p < 0.05, ***p < 0.001.

To examine homeostatic responses in deep layer PFC neurons, we developed an ex vivo acute slice paradigm (Fig. 4A; see Materials and methods for details). We found that reducing network activity by a 6-h incubation with TTX and APV reliably caused an increase in mEPSC frequency in P13–15 WT PFC neurons, with no change in mEPSC amplitude (Fig. 4B), nor any impact on mIPSCs (Supplementary Fig. 3). When we tested *Chd8*^*+/–*^ PFC neurons with the same paradigm, they failed to respond (Fig. 4B). Interestingly, we were unable to elicit plasticity through treatment with TTX alone (Fig. 4B), or with APV alone (Supplementary Fig. 3), suggesting a mechanism dependent on blocking NMDA receptors from stochastically released glutamate in the absence of neuronal firing. Spontaneous neurotransmission has previously been shown to induce homeostatic plasticity via local translation mechanisms in dissociated hippocampal neurons [42, 43]. Intriguingly, we found that the increase in mEPSC frequency seen in PFC neurons was abolished by the protein translation inhibitor anisomycin (Fig. 4C), indicating that this response is also translation dependent. We were unable to elicit changes to inhibitory transmission with TTX alone (Fig. 4D); therefore, we trialed a TTX plus Gabazine (GZ) treatment in order to block stochastic GABA transmission. This had no effect on WT neurons but triggered an abnormal response in *Chd8*^*+/–*^ neurons, resulting in a significant increase in mIPSC frequency with no change in amplitude (Fig. 4D) nor any effect on mEPSCs (Supplementary Fig. 3). Together these results show that reduced *Chd8* expression can disrupt the regulation of homeostatic plasticity in the PFC.

## Discussion

The results presented here show that reduced expression of *Chd8* significantly alters synaptic development within the mouse PFC in a highly dynamic, stage-specific manner that acts differentially within individual cell types to produce contrasting changes in excitatory and inhibitory synaptic transmission, in turn affecting neuronal output. Furthermore, *Chd8*^*+/–*^ neurons are unable to adequately retune excitatory synaptic transmission to respond to reductions in spontaneous neurotransmission, instead showing inappropriate increases in the level of inhibitory synaptic transmission.

We specifically found alterations in presynaptic function, with reduced probability of release, in excitatory synapses onto deep layer PFC neurons. This is interesting in light of recent data indicating that *Drosophila* neurons hypomorphic for the *Chd8* homolog *kismet* show presynaptic glutamatergic transmission deficits due to disruption to synaptic vesicle recycling [44]. For inhibitory synapses, the increased frequency of mIPSCs could be at least partially be explained by an increased number of inputs on to basal dendrites. Although this differential effect on basal dendrites, compared with apical ones, does not appear to correspond to compartment-specific targeting for any known interneuron subtype [45], apical and basal dendrites do receive differential input in sensory cortex [46]. It is therefore possible that this may reflect a response to differences in functional inputs.

Modeling ASD-relevant behavior in mice presents significant challenges, and for *Chd8*^*+/–*^ mouse models results have been highly variable, with some studies suggesting social deficits [33] and anxiety behaviors [8] while others have found minimal phenotypes in these behaviors [31, 32]. Furthermore, there is evidence from studies examining rodent models of Fragile X syndrome (FXS) that behavioral phenotypes can vary significantly between mice, rats, and humans while molecular and cellular alterations are relatively consistent [47, 48]. Given these issues, and in the absence of consistent behavioral alterations in the context of *Chd8* haploinsufficiency, we have therefore focused on cellular phenotypes. While our electrophysiological synaptic recordings will be biased against detecting inputs from the most distal regions of the neuron due to attenuation and inevitable space clamp issues inherent to the technique, we have complemented this with structural synaptic imaging as well as assessment of presynaptic function and neuronal output, with consistent changes across genotypes. Two previous studies have reported alterations to miniature postsynaptic currents in *Chd8*^*+/–*^ mouse models concurrent with some altered behaviors. In contrast to our findings, Platt et al. found no change in excitatory transmission within the nucleus accumbens of adult mice and reported reduced inhibitory transmission [8]. Jung et al. examined the upper layers of the cortex and hippocampus of juvenile animals with a heterozygous, frameshift point mutation of *Chd8*, finding no changes to synaptic transmission in cortex, and alterations specifically to inhibitory transmission in hippocampus [7]. These discrepancies likely result from two key differences between our studies: the brain regions in which synaptic transmission was surveyed and the developmental stage of the animals. We have provided a thorough characterization of the impact of reduced *Chd8* expression on E:I balance within pyramidal neurons of the deep layers of the PFC, a cell type and brain region that are highly relevant to ASD etiology [34, 35]. It is clear that the impact of ASD-risk gene mutation varies critically depending on brain region. For example, plasticity phenotypes, E:I imbalances and signaling pathway alterations (e.g., mTOR, ERK) in the *Fmr1*^*–/y*^ mouse model for FXS are substantially different between cortex (including some evidence from PFC) and hippocampus [48]. A recent meta-analysis of CHD8 transcriptome and CHIP-seq studies found that while CHD8 genomic interactions were relatively consistent, there was more variability in transcriptional changes, including across different brain regions [49], perhaps offering a partial clue to these regional differences. While further exploration of these differences will be an active area for the field, it remains clear that it is important to examine specific ASD-relevant brain regions. In addition, we have gone beyond previous studies by examining synaptic transmission across neurodevelopment, showing that changes to E:I balance are highly dynamic and dependent on the age of the animals. Strikingly, our study has revealed a key developmental window during the adolescence (P14-P20) of *Chd8*^*+/–*^ mice where E:I balance is substantially reduced, characterized by decreased excitatory and increased inhibitory synaptic transmission, and outside of which synaptic transmission is relatively similar to WT animals. Such developmental stage-specific changes to synaptic transmission have been reported in a *Shank3B*^*–/–*^ mouse [11], which together with our work highlights that a longitudinal examination of synaptic transmission is necessary to fully understand how E:I balance is impacted at different critical time periods in animal models of ASD.

By utilizing conditional Cre drivers we have demonstrated that cell-type-specific reduction of *Chd8* expression is sufficient to alter excitatory and inhibitory synapses in these cell types, providing new insight into how reduced *Chd8*^*+/–*^ expression impacts synaptic development and function. Our findings indicate that synaptic transmission phenotypes do not emerge through a global reduction in *Chd8* expression across neural circuits but rather result from discrete mechanisms arising within individual neuronal subtypes. Specifically, changes to inhibitory transmission are driven entirely by the reduction of *Chd8* expression in interneurons, while for excitatory synapses the reduction in mEPSC frequency appears to be the core, cell-autonomous phenotype with other changes possibly resulting from a complex interplay of different cellular responses. In addition, this excitatory phenotype arises following conditional deletion of a *Chd8* copy in postmitotic neurons, suggesting that it is not dependent on alterations occurring during early development. It is also interesting to note that the impact of reduced *Chd8* expression on inhibitory synaptic changes is to a significant extent structural, affecting synaptic densities, while our data are suggestive of primarily functional effects on excitatory neurotransmission. Determining how these potentially distinct mechanisms operate in different neuronal subtypes will be an essential avenue for future research.

The PFC is not amenable to major sensory deprivation paradigms as can be done in vivo for sensory cortical areas. We therefore developed a novel and robust assay in this study that utilizes global pharmacological blockade of AP firing and spontaneous neurotransmission to induce homeostatic synaptic responses in ex vivo slices of PFC. Using this assay, we provide the first experimental evidence for highly dysregulated homeostatic plasticity mechanisms in the PFC of *Chd8*^*+/–*^ mice. Specifically, *Chd8*^*+/–*^ neurons displayed a blunted ability to induce compensatory changes in excitatory synaptic transmission and abnormal, inappropriate changes to inhibitory synaptic transmission.

Interestingly, blockade of AP firing alone was found to be insufficient to induce plasticity changes in our paradigm, suggesting a mechanism that is dependent upon the blockade of spontaneous neurotransmitter release. Induction of homeostatic responses due to spontaneous, or miniature, transmission has been described in other neuronal cell types [50]. Rapid homeostatic scaling, although affecting mEPSC amplitude as opposed to frequency, has been previously described for the blockade of spontaneous glutamate transmission through NMDA receptors in cultured hippocampal neurons [51]. However, we found that NMDA receptor antagonist alone was unable to induce homeostatic responses, meaning that the combined blockade of neuronal firing and spontaneous release are required for induction in our paradigm. Given the relatively short time frame for induction of this response (necessary for acute ex vivo slice work), it is possible that blocking spontaneous transmission accelerates the homeostatic response [43]. We also describe the novel finding of abnormal plasticity changes in the *Chd8*^*+/–*^ neurons being initiated through the blockade of spontaneous inhibitory GABA transmission. Very little is known regarding a potential role for spontaneous GABAergic transmission in homeostatic responses, although inhibiting excitatory GABA release in chick spinal cord has previously been shown to induce scaling [52]. Intriguingly, it has been suggested that, at least for synaptic scaling, separate mechanisms may operate at excitatory and inhibitory synapses [53]. Despite differences in preparation and cell type from earlier work [43], we find that the homeostatic responses described here are also dependent on protein translation. Key mediators in spontaneous release-driven homeostatic responses seen previously in hippocampal neurons have been identified [42], and while further investigation into the signaling pathways involved in our paradigm will be necessary, this finding potentially opens up new therapeutic targets.

A recent study examining four separate ASD mouse models displaying altered E:I balance within the sensory cortex showed that these changes acted to stabilize synaptic depolarization and spiking and that the mice showed little to no change in sensory-evoked activity as a result [21]. They therefore proposed that changes to E:I balance may be the end point of homeostatic compensatory mechanisms acting to stabilize circuit excitability rather than a causal mechanism of ASD [21]. Our results are distinct from this study as we show that homeostatic plasticity mechanisms themselves can be profoundly dysregulated in the *Chd8*^*+/–*^ ASD model, and that the synaptic E:I imbalance seen in *Chd8*^*+/–*^ PFC neurons is associated with the predicted effect on neuronal firing. It should be noted that all the models examined in the study from Antoine et al. displayed increased E:I ratios, whereas we find that *Chd8*^*+/–*^ mice show a profoundly decreased E:I balance. Therefore, different mechanisms may be acting to alter cortical circuitry, perhaps indicative of distinct pathophysiological subgroups. Our findings are in agreement with a recent report showing that homeostatic plasticity mechanisms are defective in neuronal cultures with reduced *Shank3* expression and in primary visual cortex following eyelid suture in *Shank3* knockout mice [20]. We now provide evidence that homeostatic responses are both insufficient and aberrant in an ASD model within neuronal circuitry more directly relevant to ASD pathology. Therefore, we propose that dysregulation of homeostatic plasticity contributes to the establishment of a reduced E:I balance in the PFC of *Chd8*^*+/–*^ mice (Fig. 4E), thereby providing a potential mechanism through which mutation of *CHD8* can impair the function of ASD-relevant circuits.

## Supplementary information

Supplementary Information
