# Supplementary material for: Cell-type-specific synaptic imbalance and disrupted homeostatic plasticity in cortical circuits of ASD-associated Chd8 haploinsufficient mice
Source: Mol Psychiatry. 2021 Apr 9;26(7):3614–24. doi: 10.1038/s41380-021-01070-9 (PMC8505247; doi:10.1038/s41380-021-01070-9)
Supplement: Supplementary file 1 — Supplementary Information [file 41380_2021_1070_MOESM1_ESM.docx]

**Supplementary Information**

**Cell-type-specific synaptic imbalance and disrupted homeostatic plasticity in cortical circuits of ASD-associated *Chd8* haploinsufficient mice**

**Supplementary Materials and Methods**

**Mice and genotyping**

Experimental *Chd8^+/-^* mice were produced by crossing *Chd8^+/-^* with C57BL/6J mice, ensuring equal paternal and maternal inheritance of the *Chd8^-^* allele. For all genotyping, genomic DNA was extracted from ear or tail samples using the HotSHOT method. Primers used were:

*Chd8^flox^* primers (Forward (F) = GCC GAG GGG ATG AGG ATA TTT AGG, Reverse (R) = GGT ACA TAT GCC TTA AAA ATC AGG CCC AG) yield a WT band of 211bp and *Chd8^flox^* band of 276bp. *Chd8^-^* primers (F = CCC ACA TCA AGT GGC TGT AA, R = GGT AGG GAA GCA GTG TCC AG) yielded a WT band of 1.1kb and a *Chd8^-^* band of 395bp. *Cre* primers (F = CCT GGA AAA TGC TTC TGT CCG, R = CAG GGT GTT ATA AGC AAT CCC) yielded a *Cre* band of 390bp. *EGFP* primers (CCT ACG GCG TGC AGT GCT TCA GC, R = CGG CGA GCT GCA CGC TGC GTC CTC) yielded an *EGFP* band of approximately 350bp.

**Electrophysiology**

*Acute brain slice preparation*

Mice were anaesthetised by isofluorane inhalation and immediately decapitated. 300 µm thick anterior coronal slices containing the prefrontal cortex (PFC) were prepared from dissected brains using a VT1000S vibratome (Leica). Brains remained in ice-cold cutting solution (240 mM sucrose, 5 mM KCl, 1.25 mM Na_2_PO­_4_, 2 mM MgSO_4_, 1 mM CaCl_2_, 26 mM NaHCO_3_ and 10 mM D-glucose) equilibrated with 95% O_2_/5% CO_2_ throughout the dissection and slicing procedures. Slices were allowed to recover in room-temperature artificial cerebrospinal fluid (ACSF; 124 mM NaCl, 5 mM KCl, 1.25 mM Na_2_HPO_4_, 2 mM MgSO_4_, 2 mM CaCl_2_, 26 mM NaHCO_3_ and 20 mM D-glucose) equilibrated with 95% O_2_/5% CO_2_ for one hour before recording. For adult mice (P55-P60) an N-methyl-D-glucamine (NMDG) cutting solution (92 mM NMDG, 92 mM HCl, 2.5 mM KCl, 1.2 mM Na H_2_PO_4_, 30 mM NaHCO_3_, 20 mM HEPES, 25 mM glucose, 5 mM sodium ascorbate, 2 mM thiourea, 3 mM sodium pyruvate, 10 mM MgSO_4_, 0.5 mM CaCl_2_) was used followed by Na^+^ reintroduction ^1^ to aid neuronal survival.

*Whole-cell patch clamp electrophysiology*

Whole-cell patch clamp recordings were taken from cortical pyramidal neurons located within layers V and VI of the PFC. Slices were continuously perfused with ACSF equilibrated with 95% O_2_/5% CO_2_, held in position using a platinum wire harp and visualised using an Olympus BX51WI microscope and Rolera Bolt camera under a 40x water-dipping objective. Neurons with a large diameter and pyramidal-shaped soma within the correct cortical region were targeted for recordings. This method was presumed to largely exclude inhibitory interneurons, which are known to have small, round somas and represent a minority of cells within the cortex (<20%). All recordings were performed at room temperature using a Patch clamp EPC 10 USB amplifier and PatchMaster software (HEKA) with signals filtered at 10 kHz and sampled at 50 kHz. All traces were recorded and analyzed blind to genotype.

*Miniature postsynaptic current recordings*

For miniature postsynaptic current recordings 1 μM tetrodotoxin (TTX, Tocris) was added to the ACSF to prevent action potential firing. Additionally, either 10 μM SR-95531 (Gabazine, Tocris) or 10 µM 2,3-dihydroxy-6-nitro-7-sulfamoyl-benzo[f]quinoxaline (NBQX, Tocris) and 25 µM (2*R*)-amino-5-phosphonovaleric acid (D-APV, Tocris) were added to isolate mEPSCs or mIPSCs respectively. Borosilicate glass electrodes (3-5 MΩ resistance) were filled with K-gluconate internal solution (135 mM K-gluconate, 10 mM KCl, 10 mM HEPES, 1 mM MgCl2, 2 mM Na-adenosine triphosphate (Na_2_ATP) and 0.4 mM Na-guanosine triphosphate (Na_3_GTP)) for mEPSC recordings while a Cl^-^-loaded internal solution (150 mM CsCl, 1.5 mM MgCl_2_, 0.5 mM EGTA, 10 mM HEPES, 4 mM Na_2_ATP, 0.4 mM Na_3_GTP) was used for mIPSC recordings. Three 60-second traces of spontaneous activity (consisting of 60x 1 second sweeps) were recorded for each neuron with membrane potential clamped at -70 mV from which the average mEPSC/mIPSC frequency and amplitude were determined. A square voltage-step pulse (± 10 mV for 10ms) was recorded before and after each trace to determine series resistance. Any cell determined to have series resistance values > 20 MΩ or whose series resistance varied by >20% over the course of recording, were excluded from further analysis. The resulting traces were analysed using MiniAnalysis Program 6.0.3 software (Synaptosoft). The sex distribution of recordings was as follows: P5 mEPSCs = 3 WT males, 3 Chd8+/- males, 4 WT females and 3 Chd8+/- females; P14 mEPSCs = 3 WT males, 3 Chd8+/- males, 3 WT females and 3 Chd8+/- females; P20 mEPSCs = 3 WT males, 4 Chd8+/- males, 3 WT females and 4 Chd8+/- females; P55-60 mEPSCs = 3 WT males, 3 Chd8+/- males, 3 WT females and 3 Chd8+/- females; P5 mIPSCs = 3 WT males, 4 Chd8+/- males, 3 WT females and 3 Chd8+/- females; P14 mIPSCs = 3 WT males, 3 Chd8+/- males, 3 WT females and 4 Chd8+/- females; P20 mIPSCs = 4 WT males, 3 Chd8+/- males, 4 WT females and 4 Chd8+/- females; P55-60 mIPSCs = 5 WT males, 3 Chd8+/- males, 3 WT females and 3 Chd8+/- females.

*Intrinsic cell properties recordings*

All intrinsic cell properties were recorded in standard ACSF using K-gluconate internal solution. Upon cell break in, resting membrane potential (RMP) was recorded immediately to minimise the impact of dialysis with internal solution. RMP was determined by switching to current clamp mode and injecting 0 current then compensating for liquid junction potential (14.7 mV). In voltage clamp mode a square voltage-step pulse (± 10 mV for 10 ms) was then administered. Whole-cell capacitance was calculated as the integral of the resulting capacitive transient and membrane resistance (*Rm*) was calculated from the resulting current step according to Ohm’s law (*Rm = V/I*). The frequency of action potential firing versus current stimulus (*f-I* curves) was then measured in current clamp using a 12-step 1 second current injection protocol starting at -80 pA and increasing in 50 pA intervals. To assess action potential characteristics, a 10-step protocol with a finer 5 pA interval was used in order to elicit voltage traces containing a single action potential. The amplitude, width and firing threshold of this single event were then determined using a custom-written MATLAB script (MathWorks).

*Paired-pulse ratio recordings*

All paired-pulse ratio (PPR) recordings were performed in standard ACSF using K-gluconate internal solution. A parallel bipolar electrode (FHC) connected to a DS3 constant current isolated stimulator (Digitimer) was placed in the deep layers of the PFC in a superficial position relative to the neurons targeted for recording. 500 µs depolarising stimuli of sequentially increasing amplitude were administered to determine the minimum amplitude required to evoke an EPSC (Eθ). PPR was then recorded from each cell in three separate sweeps. Each sweep consisted of two 500 µs stimuli with amplitudes of 1.5 x Eθ (typically 0.8-1.2 mA) administered 25 ms apart to elicit two EPSCs (*E_1_* and *E_2_*) and was followed by a 1-minute recovery period. The amplitudes of *E_1_* and *E_2_* were determined using a custom-written MATLAB script (Mathworks), with *E_1_* calculated relative to initial baseline and *E_2_* relative to an extrapolated curve fitted to *E_1_.* Paired-pulse ratio was calculated for each sweep as *E_2_ / E_1_* to generate a mean value for each recorded cell.

**Sholl Analysis**

Mice were sacrificed at postnatal day 22 (P22) by rising CO2 concentration. Brains were dissected and Golgi-Cox stained according to the FD Rapid GolgiStain Kit (FD Neurotechnologies) protocol. Before the stain development step, impregnated brains were embedded in 4% low-melting point agarose (Thermo Scientific) and cut into 100 μm coronal sections using a VT1000S vibratome (Leica). Brightfield Z-stacks of stained neurons were captured using an Eclipse Ti microscope (Nikon) under a 20x objective. Dendritic branching patterns were reconstructed in three dimensions from Z-stacks by manually tracing the basal dendritic tree of layer V/VI pyramidal projection neurons using the Image J plugin Simple Neurite Tracer^2^. The in-built Sholl analysis function of Simple Neurite Tracer was used to perform the analysis with a radius step size of 5 microns.

**Principal Component Analysis**

A total of 22 parameters was used for cluster analysis (CA) and principal component analysis (PCA). This included passive membrane properties (input resistance and capacitance), single spike properties measured at spiking threshold stimulus (AP threshold, rheobase, AP maximum voltage, AP amplitude, post-AP AHP, width half-height, maximum AP rise, AP latency), and spike train properties measured at +150pA from rheobase (number of APs, first spike amplitude, AP steady state frequency, AP instantaneous frequency, burstiness, AP frequency adaptation, and AP amplitude adaptation). For each spike train property except AP amplitude adaptation, the slope of the change with each +50pA current step was also included.

All CA and PCA analysis was performed in the Scikit-learn plugin for Python^3^. For CA, the data was clustered with hierarchical clustering using Ward’s method. For CA, the data was clustered with hierarchical clustering using Ward’s method. The number of clusters was decided by Scikit-learn agglomerative clustering algorithm (linkage threshold set at 13) and confirmed by locating the maximal derivative of the sorted linkage distance and using it as a cut-off value^4^. For PCA, the data was transformed to a single scale using z-score standardisation for each parameter. Missing values were replaced using the Scikit-learn impute function based on the Euclidean distance to the 3 nearest neighbours in coordinate space.

**Synapse Analysis**

*Immunohistochemistry*

P14 and P20 *Chd8^+/-^;Thy1-GFP-M* mice were deeply anaesthetised with Euthatal (Merial) and transcardially perfused with 5 ml phosphate buffered saline (PBS) followed by 5 ml 4% paraformaldehyde (PFA). Brains were dissected and postfixed overnight in 4% PFA before being embedded in 4% low-melting point agarose (Thermo Scientific) and cut into 100 μm coronal sections using a Leica VT 1000S vibratome. Sections containing the PFC were permeabilized in PBS + 1% Triton-X100 (PBS-T) for 4 hours then placed in block solution (3% Bovine Serum Albumin (BSA), 10% Foetal Bovine Serum (FBS), 0.2 M glycine, in PBS-T) overnight. Next, the sections were incubated at 4°C for 3 days in primary antibodies specific for green fluorescent protein (GFP; 1:1000, chicken anti-GFP, Abcam) and vesicular GABA transporter (VGAT; 1:1000, rabbit anti-VGAT, Synaptic Systems) diluted in block solution. Sections were washed with PBS-T then incubated overnight at room temperature in secondary antibodies (goat α-chicken Fluor488 and goat α-rabbit Fluor568, 1:2000, Alexa) diluted in block solution. Sections were washed with PBS-T and PBS then mounted onto glass slides with Mowiol (Sigma).

*Image acquisition and analysis*

Z-stack images were taken of dendrites (and associated VGAT staining) from GFP+ layer V/VI projection neurons within the PFC using either a Zeiss LSM 800 or Nikon A1R Point-Scanning Confocal Microscope under 63x or 100x oil immersion objectives. A single secondary apical and basal dendrite were imaged per neuron and a minimum of 4 neurons were imaged per animal. The dendrites and the spines were reconstructed using the filament tracer tool within IMARIS software (BitPlane) from which dendrite length and spine density were quantified. The Spots function was then used to detect the VGAT staining puncta within 1µm of the dendritic shaft. The mean VGAT puncta size was calculated for each animal and used as the estimated XY diameter during spot detection so as to normalise synapse detection between animals. All imaging and analysis were performed blind to sample genotype.

**Supplementary Figures**

**
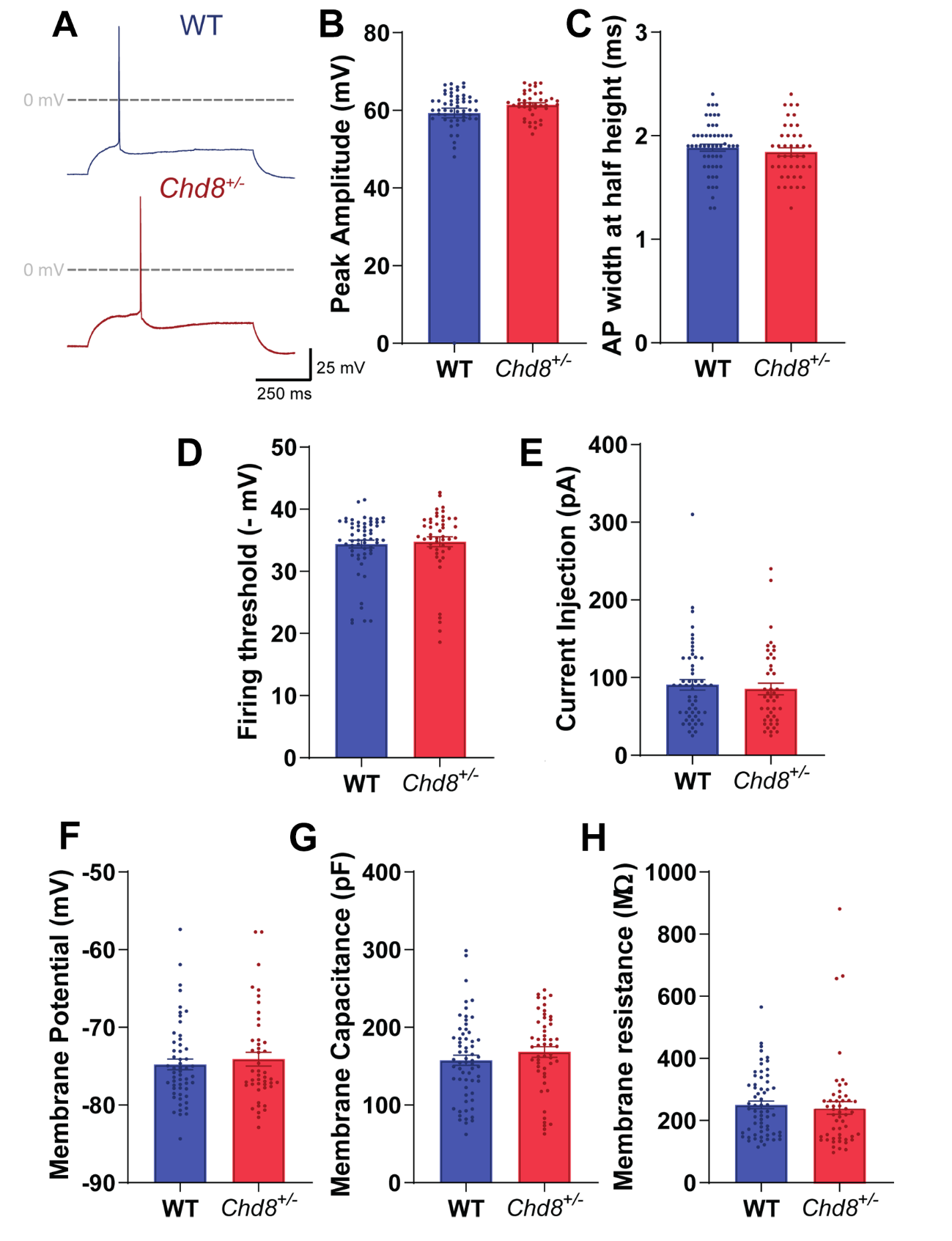
Supplementary Figure 1. Intrinsic and passive membrane properties in *Chd8^+/-^* neurons**

(**A**) Representative individually-elicited APs. *Chd8^+/-^* neurons showed no difference in AP amplitude (**B**), width at half height (**C**), firing threshold (**D**) or rheobase (**E**). Passive membrane properties: *Chd8^+/-^* neurons showed no difference in resting membrane potential (**F**), whole-cell capacitance (**G**) or membrane resistance (**H**).


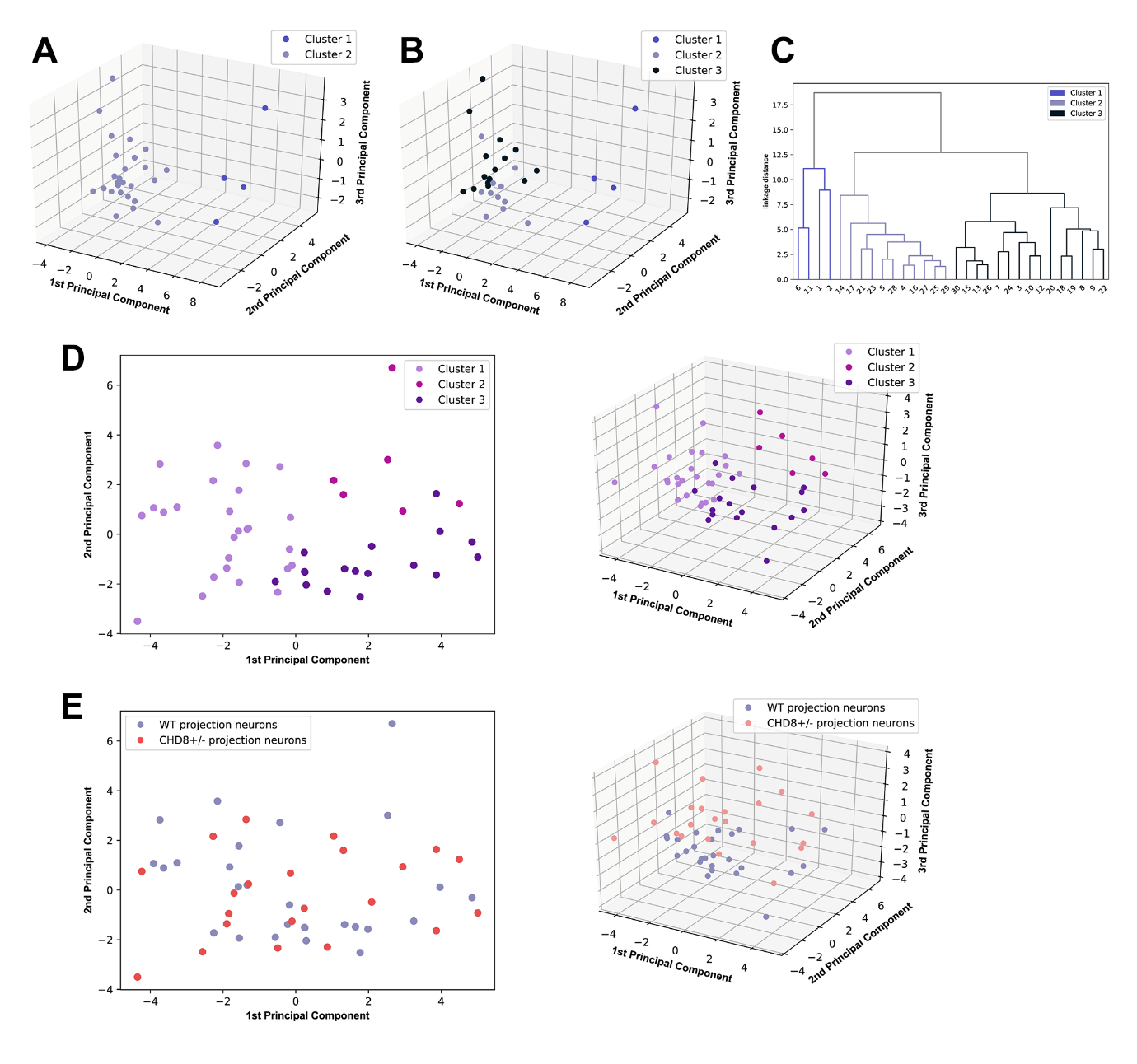


**Supplementary Figure 2. Principal component analysis and hierarchical clustering of electrophysiological data**

Intrinsic and passive membrane properties from WT (**A-C**) or combined WT and *Chd8^+/-^* (**D-E**) were analyzed. (**A**) First 3 principal components for WT data. Ward’s hierarchical clustering (as per Methods) extracts only two clusters. (**B**) First 3 principal components colour coded when clustering manually constrained to extract 3 clusters, and seen on dendrogram (**C**). However, the two main clusters (clusters 2 and 3) do not correspond to known deep layer excitatory neuronal subgroups – see Supplementary Tables 1 and 2. (**D**) Graphs showing 2 PC (*left*) and 3 PC (*right*) for combined WT and *Chd8^+/-^* data, clustering as per methods. (**E**) As for (**D**) but colour coded for WT vs *Chd8^+/-^*. Genotype does not correspond to clusters.


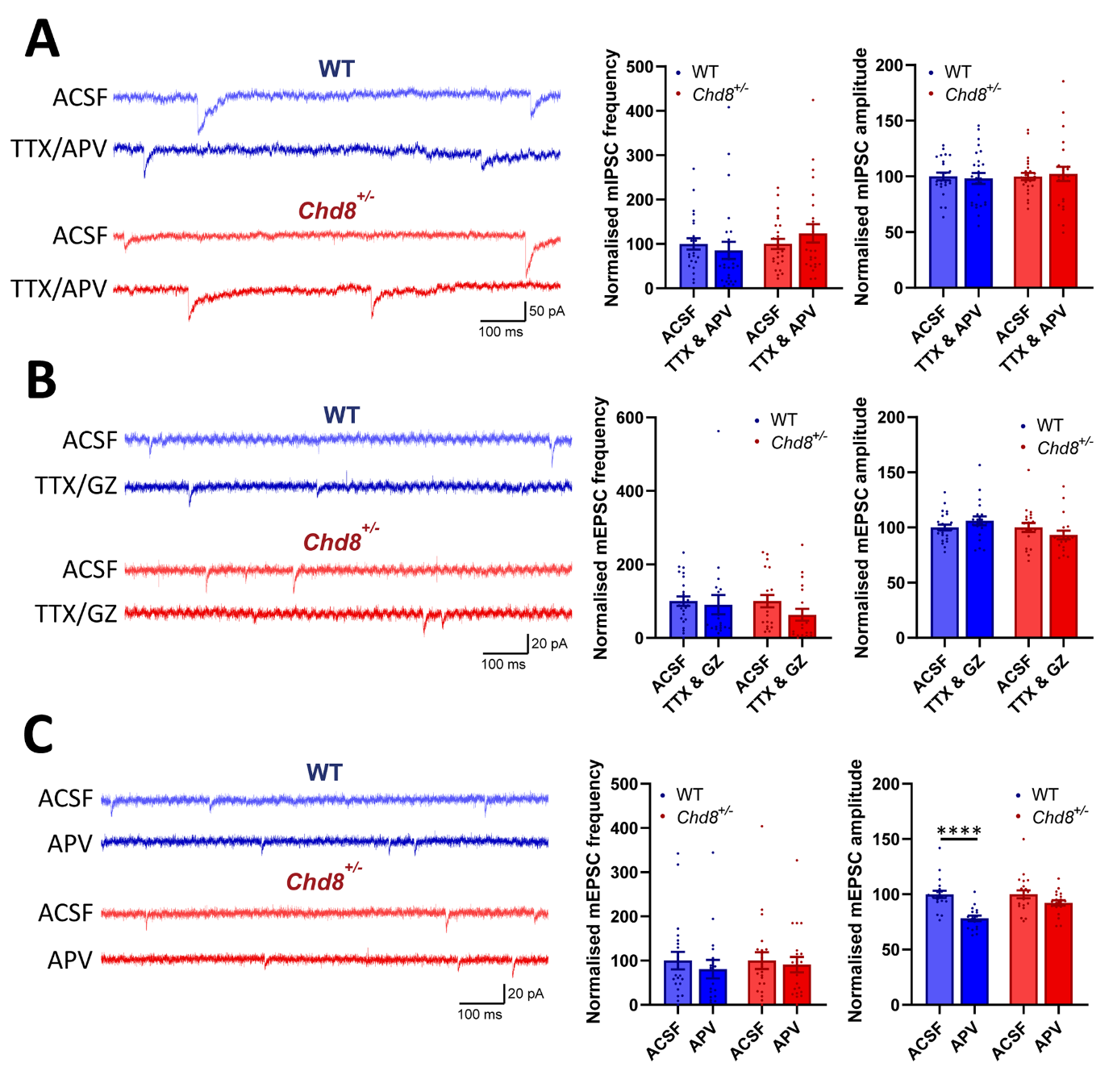


**Supplementary Figure 3. Additional activity modulation data**. (**A-C)** Representative mIPSC (**A**) or mEPSC (**B,C**) recordings from WT (*blue*) and *Chd8^+/-^* (*red*) neurons incubated for 6 hours in ACSF or drugs (*left*) alongside quantifications of frequency (*middle*) and amplitude (*right*). (**A**) Effect of incubation with TTX+APV for 6 hours on mISPCs. (**B**) Effect of incubation with TTX+GZ for 6 hours on mESPCs. (**C**) Effect of incubation with APV alone for 6 hours on mEPSCs.

**Supplementary Tables:**

**Supplementary Table 1:** *Descriptive statistics and results of pairwise analyses*

| **Pairwise Analyses (Mann-Whitney U & t-test)** | | | | | | |
| --- | --- | --- | --- | --- | --- | --- |
| **Comparison (Units)** | **Genotype** | **N (Neurons (animals))** | **Mean** | **S.E.M (±)** | **Test** | **P** |
| **P20 mEPSC frequency (Hz)** | WT | 38 (6) | 1.023 | 0.2 | MW-U | **0.0027** |
|  | *Chd8^+/-^* | 44 (8) | 0.46 | 0.071 |  |  |
| **P20 mEPSC amplitude (pA)** | WT | 38(6) | 14.78 | 0.88 | MW-U | **< 0.0001** |
|  | *Chd8^+/-^* | 45 (8) | 10.87 | 0.54 |  |  |
| **P20 mIPSC frequency (Hz)** | WT | 62 (8) | 3.97 | 0.28 | t-test | **0.0009** |
|  | *Chd8^+/-^* | 55 (7) | 5.73 | 0.43 |  |  |
| **P20 mIPSC amplitude (pA)** | WT | 62 (8) | 43.71 | 1.011 | MW-U | **0.14** |
|  | *Chd8^+/-^* | 55 (7) | 41.39 | 1.33 |  |  |
| **AP amplitude (mV)** | WT | 54 (8) | 59.33 | 1.25 | MW-U | **0.2605** |
|  | *Chd8^+/-^* | 42 (6) | 61.44 | 0.52 |  |  |
| **AP width-at-half-height (ms)** | WT | 54 (8) | 1.89 | 0.034 | t-test | **0.4228** |
|  | *Chd8^+/-^* | 42 (6) | 1.84 | 0.04 |  |  |
| **Rheobase (pA)** | WT | 54 (8) | 90.67 | 6.99 | MW-U | **0.473** |
|  | *Chd8^+/-^* | 42 (6) | 85.36 | 7.54 |  |  |
| **AP firing threshold (mV)** | WT | 54 (8) | 34.38 | 0.62 | MW-U | **0.3814** |
|  | *Chd8^+/-^* | 42 (6) | 34.77 | 0.79 |  |  |
| **Resting membrane potential (mV)** | WT | 55 (8) | -74.77 | 0.7 | MW-U | **0.7742** |
|  | *Chd8^+/-^* | 46 (6) | -74.09 | 0.88 |  |  |
| **Whole-cell capacitance (pF)** | WT | 62 (8) | 157.6 | 6.62 | t-test | **0.2677** |
|  | *Chd8^+/-^* | 51 (6) | 168.4 | 6.93 |  |  |
| **Membrane Resistance (MΩ)** | WT | 62 (8) | 250.4 | 12.8 | MW-U | **0.1505** |
|  | *Chd8^+/-^* | 51 (6) | 241 | 20.39 |  |  |
| **P5 mEPSC frequency (Hz)** | WT | 28 (7) | 0.082 | 0.016 | MW-U | **0.3508** |
|  | *Chd8^+/-^* | 32 (4) | 0.12 | 0.028 |  |  |
| **P14 mEPSC frequency (Hz)** | WT | 34 (5) | 0.48 | 0.078 | MW-U | **0.4954** |
|  | *Chd8^+/-^* | 38 (4) | 0.36 | 0.041 |  |  |
| **P55-60 mEPSC frequency (Hz)** | WT | 39 (6) | 1.75 | 0.29 | MW-U | **0.1224** |
|  | *Chd8^+/-^* | 29 (5) | 1.072 | 0.25 |  |  |
| **P5 mEPSC amplitude (pA)** | WT | 27 (7) | 13.38 | 1.12 | MW-U | **0.7454** |
|  | *Chd8^+/-^* | 32 (4) | 13.14 | 0.68 |  |  |
| **P14 mEPSC amplitude (pA)** | WT | 34 (5) | 14.84 | 1.002 | MW-U | **0.0004** |
|  | *Chd8^+/-^* | 38 (4) | 11.26 | 0.5 |  |  |
| **P55-60 mEPSC amplitude (pA)** | WT | 39 (6) | 13.4 | 0.41 | MW-U | **0.5296** |
|  | *Chd8^+/-^* | 29 (5) | 14.04 | 0.91 |  |  |
| **P5 mIPSC frequency (Hz)** | WT | 40 (5) | 0.033 | 0.0058 | MW-U | **0.345** |
|  | *Chd8^+/-^* | 40 (7) | 0.021 | 0.02 |  |  |
| **P14 mIPSC frequency (Hz)** | WT | 41 (6) | 1.62 | 0.21 | MW-U | **0.6239** |
|  | *Chd8^+/-^* | 38 (7) | 1.76 | 0.23 |  |  |
| **P55-60 mIPSC frequency (Hz)** | WT | 38 (6) | 6.67 | 0.79 | MW-U | **0.8018** |
|  | *Chd8^+/-^* | 26 (5) | 6.43 | 0.91 |  |  |
| **P5 mIPSC amplitude (pA)** | WT | 34 (5) | 29.88 | 2.035 | MW-U | **<0.0001** |
|  | *Chd8^+/-^* | 37 (7) | 18.4 | 1.14 |  |  |
| **P14 mIPSC amplitude (pA)** | WT | 41 (6) | 34.78 | 1.18 | t-test | **0.008** |
|  | *Chd8^+/-^* | 38 (7) | 42.15 | 1.72 |  |  |
| **P55-60 mIPSC amplitude (pA)** | WT | 38 (6) | 27.15 | 1.23 | MW-U | **0.0328** |
|  | *Chd8^+/-^* | 26 (5) | 32.36 | 1.955 |  |  |
| **P14 apical spines (per 10 µm)** | WT | 29 (5) | 7.12 | 0.44 | MW-U | **0.5595** |
|  | *Chd8^+/-^* | 30 (6) | 7.074 | 0.44 |  |  |
| **P14 basal spines (per 10 µm)** | WT | 26 (5) | 5.054 | 0.44 | MW-U | **0.0613** |
|  | *Chd8^+/-^* | 32 (6) | 6.122 | 0.35 |  |  |
| **P14 apical VGAT puncta (per 10 µm)** | WT | 29 (5) | 2.55 | 0.31 | MW-U | **0.2823** |
|  | *Chd8^+/-^* | 30 (6) | 3.68 | 0.71 |  |  |
| **P14 basal VGAT puncta (per 10 µm)** | WT | 26 (5) | 2.72 | 0.31 | MW-U | **0.2608** |
|  | *Chd8^+/-^* | 32 (6) | 2.7 | 0.58 |  |  |
| **P20 apical spines (per 10 µm)** | WT | 28 (5) | 8.16 | 0.47 | t-test | **0.2722** |
|  | *Chd8^+/-^* | 24 (5) | 7.53 | 0.32 |  |  |
| **P20 basal spines (per 10 µm)** | WT | 27 (5) | 8.11 | 0.56 | t-test | **0.5184** |
|  | *Chd8^+/-^* | 23 (5) | 7.68 | 0.36 |  |  |
| **P20 apical VGAT puncta (per 10 µm)** | WT | 25 (5) | 3.76 | 0.52 | MW-U | **>0.9999** |
|  | *Chd8^+/-^* | 24 (5) | 3.94 | 0.65 |  |  |
| **P20 basal VGAT puncta (per 10 µm)** | WT | 24 (5) | 3.63 | 0.63 | MW-U | **0.0002** |
|  | *Chd8^+/-^* | 23 (5) | 6.89 | 1.086 |  |  |
| ***Nkx2.1* mEPSC frequency (Hz)** | *Cre^-^* | 51 (6) | 1.49 | 0.15 | MW-U | **0.7547** |
|  | *cChd8^+/--^* | 45 (5) | 1.45 | 0.15 |  |  |
| ***Nkx2.1* mEPSC amplitude (pA)** | *Cre^-^* | 51 (6) | 14.39 | 0.31 | MW-U | **0.2952** |
|  | *cChd8^+/--^* | 45 (5) | 14.34 | 0.49 |  |  |
| ***Nkx2.1* mIPSC frequency (Hz)** | *Cre^-^* | 22 (3) | 2.38 | 0.35 | MW-U | **< 0.0001** |
|  | *cChd8^+/--^* | 23 (3) | 5.069 | 0.46 |  |  |
| ***Nkx2.1* mIPSC amplitude (pA)** | *Cre^-^* | 22 (3) | 42 | 3.26 | MW-U | **0.5815** |
|  | *cChd8^+/--^* | 23 (3) | 39.85 | 1.44 |  |  |
| ***NEX* mEPSC frequency (Hz)** | *Cre^-^* | 41 (7) | 1.13 | 0.21 | MW-U | **0.0027** |
|  | *cChd8^+/--^* | 30 (4) | 0.47 | 0.064 |  |  |
| ***NEX* mEPSC amplitude (pA)** | *Cre^-^* | 41 (7) | 16.68 | 0.51 | MW-U | **< 0.0001** |
|  | *cChd8^+/--^* | 30 (4) | 19.87 | 0.49 |  |  |
| ***NEX* mIPSC frequency (Hz)** | *Cre^-^* | 38 (6) | 1.31 | 0.18 | MW-U | **0.0108** |
|  | *cChd8^+/--^* | 31 (4) | 0.86 | 0.14 |  |  |
| ***NEX* mIPSC amplitude (pA)** | *Cre^-^* | 38 (6) | 39.34 | 1.84 | MW-U | **0.2877** |
|  | *cChd8^+/--^* | 31 (4) | 36.12 | 1.51 |  |  |

**Supplementary Table 2:** *Descriptive statistics and results of multivariate analyses*

| **Multivariate Analyses (2-way ANOVA unless stated)** | | | | | | | | | |
| --- | --- | --- | --- | --- | --- | --- | --- | --- | --- |
| **Comparison (Units)** | **Genotype** | **Treatment** | **N (Neurons (animals))** | **Mean** | **S.E.M (±)** | **Variable** | **F** | **P** | **Tukey's adjusted P** |
| **Sholl Analysis** | WT | N/A | 45 (4) | N/A | N/A | Genotype | (1, 1163) = 0.39 | 0.53 | **N/A** |
|  | *Chd8^+/-^* | N/A | 24 (3) |  |  |  |  |  |  |
| ***f-I* curves** | WT | N/A | 54 (8) | N/A | N/A | Genotype | (1, 96) = 0.07 | 0.78 | **N/A** |
|  | *Chd8^+/-^* | N/A | 42 (6) | N/A | N/A |  |  |  |  |
| **Plasticity mEPSC frequency  (normalised)** | WT | ACSF | 20 (3) | 100 | 17.6 | Genotype | (1, 68) = 4.96 | 0.03 | **0.0003** |
|  |  | TTX & APV | 19 (3) | 479.9 | 112.5 |  |  |  |  |
|  | *Chd8^+/-^* | ACSF | 17 (3) | 100 | 19.08 |  |  |  | **0.93** |
|  |  | TTX & APV | 16 (3) | 189.6 | 50.59 |  |  |  |  |
|  | WT | ACSF | 21 (4) | 100 | 22.37 | Treatment | (1, 85) = 0.54 | 0.47 | **0.99** |
|  |  | TTX | 23 (4) | 109.1 | 23.18 |  |  |  |  |
|  | *Chd8^+/-^* | ACSF | 22 (4) | 100 | 19.66 |  |  |  | **0.99** |
|  |  | TTX | 23 (4) | 129.2 | 35.47 |  |  |  |  |
|  | WT | ACSF | 25 (5) | 100 | 12.6 | Treatment | (1, 81) = 5.86 | 0.02 | **0.35** |
|  |  | TTX & GZ | 21 (5) | 90.4 | 26.2 |  |  |  |  |
|  | *Chd8^+/-^* | ACSF | 20 (4) | 100 | 16.9 |  |  |  | **0.30** |
|  |  | TTX & GZ | 20 (4) | 62.92 | 16.4 |  |  |  |  |
|  | WT | ACSF | 14 (3) | 100 | 11.9 | One-way ANOVA | (2, 42) = 5.29 | 0.009 | **0.003** (ACSF vs TTX & APV) |
|  |  | TTX & APV | 16 (3) | 465.1 | 112.3 |  |  |  | **0.76** (ACSF vs TTX & APV + Anisomycin) |
|  |  | TTX & APV + Anisomycin | 15 (3) | 173 | 31.7 |  |  |  | **0.02** (TTX & APV vs TTX & APV + Anisomycin) |
| **Plasticity mEPSC amplitude  (normalised)** | WT | ACSF | 20 (3) | 100 | 4.13 | Treatment | (1, 68) = 2.62 | 0.11 | **0.84** |
|  |  | TTX & APV | 20 (3) | 92.71 | 5.06 |  |  |  |  |
|  | *Chd8^+/-^* | ACSF | 17 (3) | 100 | 4.7 |  |  |  | **0.82** |
|  |  | TTX & APV | 16 (3) | 91.39 | 5.9 |  |  |  |  |
|  | WT | ACSF | 21 (4) | 100 | 4.9 | Treatment | (1, 84) = 0.17 | 0.68 | **0.71** |
|  |  | TTX | 23 (4) | 92.65 | 4.3 |  |  |  |  |
|  | *Chd8^+/-^* | ACSF | 22 (4) | 100 | 3.3 |  |  |  | **0.36** |
|  |  | TTX | 22 (4) | 111.3 | 6.5 |  |  |  |  |
|  | WT | ACSF | 24 (5) | 100 | 2.7 | Treatment | (1, 81) = 0.01 | 0.92 | **0.62** |
|  |  | TTX & GZ | 21 (5) | 106 | 4 |  |  |  |  |
|  | *Chd8^+/-^* | ACSF | 20 (4) | 100 | 4.1 |  |  |  | **0.58** |
|  |  | TTX & GZ | 19 (4) | 93.2 | 3.9 |  |  |  |  |
|  | WT | ACSF | 14 (3) | 100 | 5.1 | One-way ANOVA | (2, 42) = 2.15 | 0.28 | **0.95** (ACSF vs TTX & APV) |
|  |  | TTX & APV | 16 (3) | 98.1 | 5 |  |  |  | **0.30** (ACSF vs TTX & APV + Anisomycin) |
|  |  | TTX & APV + Anisomycin | 15 (3) | 90.6 | 2.3 |  |  |  | **0.44** (TTX & APV vs TTX & APV + Anisomycin) |
| **Plasticity mIPSC frequency (normalised)** | WT | ACSF | 24 (5) | 100 | 12.7 | Treatment | (1, 95) = 0.07 | 0.78 | **0.92** |
|  |  | TTX & APV | 26 (5) | 85.4 | 19.4 |  |  |  |  |
|  | *Chd8^+/-^* | ACSF | 25 (5) | 100 | 11.4 |  |  |  | **0.75** |
|  |  | TTX & APV | 24 (5) | 123.7 | 20.7 |  |  |  |  |
|  | WT | ACSF | 33 (6) | 100 | 12.26 | Treatment | (1, 119) = 1.59 | 0.21 | **0.93** |
|  |  | TTX | 30 (6) | 107.6 | 16.8 |  |  |  |  |
|  | *Chd8^+/-^* | ACSF | 33 (6) | 100 | 15.1 |  |  |  | **0.62** |
|  |  | TTX | 32 (6) | 127.4 | 19.9 |  |  |  |  |
|  | WT | ACSF | 31 (6) | 100 | 11.2 | Genotype | (1, 98) = 5.21 | 0.02 | **> 0.99** |
|  |  | TTX & GZ | 29 (6) | 100.6 | 15.5 |  |  |  |  |
|  | *Chd8^+/-^* | ACSF | 21 (4) | 100 | 12.26 |  |  |  | **0.02** |
|  |  | TTX & GZ | 21 (4) | 187.1 | 34.3 |  |  |  |  |
| **Plasticity mIPSC amplitude  (normalised)** | WT | ACSF | 24 (5) | 100 | 3.5 | Treatment | (1, 95) = 0.002 | 0.96 | **0.99** |
|  |  | TTX & APV | 26 (5) | 98.2 | 4.9 |  |  |  |  |
|  | *Chd8^+/-^* | ACSF | 25 (5) | 100 | 3.3 |  |  |  | **0.99** |
|  |  | TTX & APV | 24 (5) | 102.2 | 6.3 |  |  |  |  |
|  | WT | ACSF | 30 (6) | 100 | 2.5 | Treatment | (1, 119) = 1.38 | 0.24 | **0.34** |
|  |  | TTX | 28 (6) | 112 | 7.7 |  |  |  |  |
|  | *Chd8^+/-^* | ACSF | 33 (6) | 100 | 3.6 |  |  |  | **> 0.99** |
|  |  | TTX | 32 (6) | 99.5 | 4.8 |  |  |  |  |
|  | WT | ACSF | 31 (6) | 100 | 2.4 | Treatment | (1, 98) = 0.48 | 0.49 | **> 0.99** |
|  |  | TTX & GZ | 29 (6) | 101.3 | 6.7 |  |  |  |  |
|  | *Chd8^+/-^* | ACSF | 21 (4) | 100 | 3.5 |  |  |  | **0.88** |
|  |  | TTX & GZ | 21 (4) | 106 | 7.04 |  |  |  |  |

|  | Literature | |
| --- | --- | --- |
|  | IT neurons | ET neurons |
| Input resistance (MΩ) | ↑^5–22^ | ↓ |
| Capacitance (pF) | ↓ | ↑^6,8–12,20^ |
| AP threshold (mV) | ↑^9,14,20,23^ | ↓ |
| Rheobase (pA) | (↓) | (↑)^8,11,13,20^ |
| Burstiness | ↓ | ↑^14,24^ |
| AP amplitude (mV) | (↓) | (↑)^12,20,24^ |
| Width half-height (ms) | ↑^9,20,24,25^ | ↓ |
| Frequency adaptation | (↑)^8,9,12–14,20,24,26^ | (↓) |
| AHP (mV) | (↓) | (↑)^9,14,23,25^ |

**Supplementary Table 3**. Relative differences in some of the key electrophysiological properties differing between intratelencephalic (IT) and extratelencephalic (ET) layer V projection neurons in rodent cortex as reported by the literature. ↑: value higher in the population, ↓: value lower in the population, (↑): value reported as higher in this population by most sources, (↓): value reported as lower in this population by most sources.

|  | Observed data | |
| --- | --- | --- |
|  | CL2 neurons | CL3 neurons |
| Input resistance (MΩ) | ↑ | ↓ |
| Capacitance (pF) | ↓ | ↑ |
| AP threshold (mV) | = | = |
| Rheobase (pA) | ↓ | ↑ |
| Burstiness | = | = |
| AP amplitude (mV) | = | = |
| Width half-height (ms) | ↑ | ↓ |
| Frequency adaptation | ↑ | ↓ |
| AHP (mV) | = | = |

**Supplementary Table 4**. Relative differences observed in cluster 2 (CL2) and cluster 3 (CL3) of WT deep layer projection neurons included in the study. The major WT neuron clusters identified in the study do not exhibit a clear IT or ET identity. ↑: value higher in the population, ↓: value lower in the population, =: values do not differ between populations.

|  | **PC1** | **PC2** | **PC3** | **PC4** | **PC5** | **PC6** | **PC7** | **PC8** | **PC9** | **PC10** |
| --- | --- | --- | --- | --- | --- | --- | --- | --- | --- | --- |
| Input resistance | 0.17 | -0.16 | -0.16 | 0.31 | -0.26 | -0.29 | -0.13 | 0.30 | -0.29 | 0.08 |
| Capacitance | -0.24 | 0.34 | 0.36 | -0.02 | -0.09 | -0.05 | -0.09 | -0.14 | -0.04 | -0.21 |
| Rheobase | -0.12 | 0.16 | 0.22 | 0.19 | -0.28 | -0.26 | 0.05 | -0.03 | -0.22 | -0.14 |
| AP voltage threshold | 0.07 | -0.10 | 0.00 | 0.02 | 0.16 | 0.09 | -0.31 | -0.09 | 0.07 | -0.09 |
| AP maximum voltage | 0.01 | -0.03 | 0.01 | 0.14 | -0.18 | -0.17 | -0.53 | 0.20 | -0.13 | 0.01 |
| AP amplitude | 0.00 | 0.10 | 0.06 | -0.08 | 0.11 | 0.10 | -0.19 | 0.03 | 0.08 | 0.23 |
| Afterhyperpolarisation | -0.21 | -0.06 | 0.22 | 0.00 | -0.02 | -0.01 | 0.42 | 0.37 | -0.03 | -0.44 |
| AT width at half-height | -0.15 | -0.04 | 0.33 | -0.09 | 0.14 | 0.13 | -0.52 | 0.08 | 0.09 | -0.25 |
| Maximum AP rise velocity | -0.12 | 0.19 | -0.01 | -0.24 | -0.19 | -0.02 | -0.22 | 0.09 | -0.10 | 0.20 |
| AP latency | -0.10 | -0.19 | 0.11 | 0.18 | 0.05 | -0.04 | -0.07 | -0.14 | -0.02 | -0.41 |
| First AP amplitude | 0.37 | -0.33 | -0.23 | 0.00 | -0.07 | -0.04 | -0.12 | -0.37 | -0.07 | -0.44 |
| Number of APs | -0.11 | -0.18 | -0.26 | -0.60 | -0.28 | 0.07 | 0.00 | 0.01 | -0.23 | -0.18 |
| Number of Aps - slope | -0.17 | -0.08 | -0.14 | 0.38 | 0.24 | 0.00 | -0.04 | -0.18 | 0.26 | 0.00 |
| Instantaneous frequency | 0.53 | 0.45 | 0.10 | 0.17 | -0.01 | -0.08 | 0.11 | -0.10 | -0.15 | -0.08 |
| Instantaneous frequency - slope | -0.41 | -0.01 | -0.13 | 0.19 | -0.20 | -0.21 | -0.04 | -0.31 | -0.12 | 0.11 |
| Steady state frequency | -0.15 | -0.42 | 0.03 | 0.22 | 0.07 | -0.04 | 0.09 | 0.34 | 0.03 | 0.14 |
| Steady state frequency - slope | 0.31 | -0.21 | 0.39 | -0.05 | 0.19 | 0.14 | -0.04 | 0.21 | -0.32 | 0.08 |
| Burstiness | 0.04 | -0.41 | 0.53 | -0.09 | -0.21 | -0.10 | 0.13 | -0.45 | -0.02 | 0.35 |
| Burstiness - slope | 0.24 | -0.05 | 0.12 | -0.10 | -0.44 | -0.23 | -0.03 | 0.18 | 0.74 | -0.07 |
| AP frequency adaptation | 0.00 | 0.00 | 0.00 | 0.33 | -0.50 | 0.80 | 0.00 | 0.00 | 0.00 | 0.00 |
| AP frequency adaptation - slope | 0.00 | 0.00 | 0.00 | 0.00 | 0.00 | 0.00 | 0.00 | 0.00 | 0.00 | 0.00 |
| AP amplitude adaptation | 0.00 | 0.00 | 0.00 | 0.00 | 0.00 | 0.00 | 0.00 | 0.00 | 0.00 | 0.00 |

**Supplementary Table 5:** Coefficients for the first 10 principal components of the PCA performed exclusively on WT data. The first 3 principal components explain 70% of the data variance, while the first 10 components explain 98% of the variance.

|  | **PC1** | **PC2** | **PC3** | **PC4** | **PC5** | **PC6** | **PC7** | **PC8** | **PC9** | **PC10** |
| --- | --- | --- | --- | --- | --- | --- | --- | --- | --- | --- |
| Input resistance | 0.26 | -0.22 | -0.21 | 0.33 | -0.22 | -0.31 | -0.14 | 0.27 | -0.32 | 0.24 |
| Capacitance | -0.32 | 0.38 | 0.32 | 0.03 | -0.23 | -0.15 | -0.07 | -0.02 | -0.18 | -0.16 |
| Rheobase | -0.03 | -0.02 | -0.09 | 0.07 | -0.20 | -0.16 | 0.20 | 0.11 | -0.09 | 0.14 |
| AP voltage threshold | -0.02 | 0.05 | 0.01 | 0.23 | -0.33 | -0.32 | -0.03 | -0.13 | -0.27 | 0.04 |
| AP maximum voltage | -0.21 | 0.16 | 0.20 | 0.06 | 0.07 | 0.01 | 0.52 | 0.08 | -0.02 | -0.06 |
| AP amplitude | -0.02 | -0.07 | 0.18 | 0.14 | 0.13 | 0.00 | 0.08 | -0.01 | 0.00 | -0.21 |
| Afterhyperpolarisation | 0.07 | 0.01 | -0.24 | 0.09 | 0.02 | -0.04 | 0.13 | -0.52 | 0.12 | 0.13 |
| AT width at half-height | 0.17 | 0.05 | -0.11 | 0.16 | 0.27 | 0.06 | -0.21 | -0.21 | 0.19 | 0.14 |
| Maximum AP rise velocity | -0.15 | 0.18 | 0.23 | -0.17 | -0.13 | 0.02 | -0.59 | 0.10 | 0.05 | 0.25 |
| AP latency | 0.08 | 0.10 | 0.02 | -0.14 | 0.09 | 0.13 | 0.16 | 0.29 | 0.13 | 0.69 |
| First AP amplitude | -0.01 | 0.18 | -0.32 | -0.22 | -0.26 | -0.03 | 0.19 | 0.21 | 0.00 | -0.04 |
| Number of APs | -0.08 | -0.10 | 0.23 | 0.19 | 0.26 | 0.04 | -0.09 | 0.51 | 0.07 | -0.10 |
| Number of Aps - slope | 0.16 | -0.22 | -0.28 | -0.28 | -0.13 | 0.08 | -0.20 | 0.24 | 0.04 | -0.47 |
| Instantaneous frequency | 0.03 | 0.06 | -0.08 | -0.50 | -0.22 | 0.15 | 0.03 | -0.09 | -0.08 | 0.04 |
| Instantaneous frequency - slope | 0.64 | 0.02 | 0.50 | -0.10 | -0.01 | 0.05 | -0.05 | -0.17 | -0.22 | -0.04 |
| Steady state frequency | 0.03 | 0.12 | 0.09 | -0.26 | -0.10 | 0.09 | 0.07 | -0.03 | -0.22 | 0.11 |
| Steady state frequency - slope | 0.42 | 0.18 | 0.01 | -0.01 | 0.13 | 0.08 | 0.27 | 0.20 | -0.22 | -0.09 |
| Burstiness | 0.06 | 0.32 | -0.06 | 0.09 | 0.08 | -0.01 | -0.12 | -0.13 | -0.12 | -0.07 |
| Burstiness - slope | -0.30 | -0.61 | 0.13 | -0.17 | 0.11 | 0.16 | 0.04 | -0.16 | -0.49 | 0.14 |
| AP frequency adaptation | -0.14 | 0.33 | -0.37 | 0.06 | 0.42 | 0.22 | -0.16 | 0.04 | -0.55 | -0.01 |
| AP frequency adaptation - slope | 0.03 | 0.07 | -0.06 | 0.04 | 0.04 | 0.00 | -0.06 | 0.02 | -0.03 | -0.03 |
| AP amplitude adaptation | 0.00 | 0.00 | 0.00 | 0.44 | -0.45 | 0.78 | 0.00 | 0.00 | 0.00 | 0.00 |

**Supplementary Table 6:** Coefficients for the first 10 principal components of the PCA performed on combined WT and Chd8^+/-^ data. The first 3 principal components explain 57% of the data variance, while the first 10 components explain 92% of the variance.

**Supplementary References**

1. Ting, J. T. *et al.* Preparation of Acute Brain Slices Using an Optimized N-Methyl-D-glucamine Protective Recovery Method. *J. Vis. Exp.* (2018). doi:10.3791/53825

2. Longair, M. H., Baker, D. A. & Armstrong, J. D. Simple Neurite Tracer: open source software for reconstruction, visualization and analysis of neuronal processes. *Bioinformatics* **27**, 2453–2454 (2011).

3. Pedregosa, F. *et al.* Scikit-learn: Machine Learning in Python. *J. Mach. Learn. Res.* **12**, 2825–2830 (2011).

4. Thorndike, R. L. Who belongs in the family? *Psychometrika* **18**, 267–276 (1953).

5. Avesar, D. & Gulledge, A. T. Selective serotonergic excitation of callosal projection neurons. *Front. Neural Circuits* **6**, (2012).

6. Anastasiades, P. G., Marlin, J. J. & Carter, A. G. Cell-Type Specificity of Callosally Evoked Excitation and Feedforward Inhibition in the Prefrontal Cortex. *Cell Rep.* **22**, 679–692 (2018).

7. Morishima, M. & Kawaguchi, Y. Recurrent Connection Patterns of Corticostriatal Pyramidal Cells in Frontal Cortex. *J. Neurosci.* **26**, 4394–4405 (2006).

8. van Aerde, K. I. & Feldmeyer, D. Morphological and Physiological Characterization of Pyramidal Neuron Subtypes in Rat Medial Prefrontal Cortex. *Cereb. Cortex* **25**, 788–805 (2015).

9. Oswald, M. J., Tantirigama, M. L. S., Sonntag, I., Hughes, S. M. & Empson, R. M. Diversity of layer 5 projection neurons in the mouse motor cortex. *Front. Cell. Neurosci.* **7**, (2013).

10. Morishima, M., Morita, K., Kubota, Y. & Kawaguchi, Y. Highly Differentiated Projection-Specific Cortical Subnetworks. *J. Neurosci.* **31**, 10380–10391 (2011).

11. Hirai, Y., Morishima, M., Karube, F. & Kawaguchi, Y. Specialized Cortical Subnetworks Differentially Connect Frontal Cortex to Parahippocampal Areas. *J. Neurosci.* **32**, 1898–1913 (2012).

12. Kim, E. J., Juavinett, A. L., Kyubwa, E. M., Jacobs, M. W. & Callaway, E. M. Three Types of Cortical Layer 5 Neurons That Differ in Brain-wide Connectivity and Function. *Neuron* **88**, 1253–1267 (2015).

13. Groh, A. *et al.* Cell-Type Specific Properties of Pyramidal Neurons in Neocortex Underlying a Layout that Is Modifiable Depending on the Cortical Area. *Cereb. Cortex* **20**, 826–836 (2010).

14. Hattox, A. M. & Nelson, S. B. Layer V neurons in mouse cortex projecting to different targets have distinct physiological properties. *J. Neurophysiol.* **98**, 3330–3340 (2007).

15. Baker, A. L., O’Toole, R. J. & Gulledge, A. T. Preferential cholinergic excitation of corticopontine neurons. *J. Physiol.* **596**, 1659–1679 (2018).

16. Gee, S. *et al.* Synaptic Activity Unmasks Dopamine D2 Receptor Modulation of a Specific Class of Layer V Pyramidal Neurons in Prefrontal Cortex. *J. Neurosci.* **32**, 4959–4971 (2012).

17. Delevich, K. *et al.* Sex and Pubertal Status Influence Dendritic Spine Density on Frontal Corticostriatal Projection Neurons in Mice. *Cereb. Cortex* **30**, 3543–3557 (2020).

18. Seong, H. J. & Carter, A. G. D1 Receptor Modulation of Action Potential Firing in a Subpopulation of Layer 5 Pyramidal Neurons in the Prefrontal Cortex. *J. Neurosci.* **32**, 10516–10521 (2012).

19. Anastasiades, P. G., Boada, C. & Carter, A. G. Cell-Type-Specific D1 Dopamine Receptor Modulation of Projection Neurons and Interneurons in the Prefrontal Cortex. *Cereb. Cortex* **29**, 3224–3242 (2019).

20. Dembrow, N. C., Chitwood, R. A. & Johnston, D. Projection-Specific Neuromodulation of Medial Prefrontal Cortex Neurons. *J. Neurosci.* **30**, 16922–16937 (2010).

21. Otsuka, T. & Kawaguchi, Y. Firing-Pattern-Dependent Specificity of Cortical Excitatory Feed-Forward Subnetworks. *J. Neurosci.* **28**, 11186–11195 (2008).

22. Kalmbach, B. E., Chitwood, R. A., Dembrow, N. C. & Johnston, D. Dendritic Generation of mGluR-Mediated Slow Afterdepolarization in Layer 5 Neurons of Prefrontal Cortex. *J. Neurosci.* **33**, 13518–13532 (2013).

23. Le Be, J.-V., Silberberg, G., Wang, Y. & Markram, H. Morphological, Electrophysiological, and Synaptic Properties of Corticocallosal Pyramidal Cells in the Neonatal Rat Neocortex. *Cereb. Cortex* **17**, 2204–2213 (2007).

24. Kasper, E. M., Larkman, A. U., Lübke, J. & Blakemore, C. Pyramidal neurons in layer 5 of the rat visual cortex. I. Correlation among cell morphology, intrinsic electrophysiological properties, and axon targets. *J. Comp. Neurol.* **339**, 459–474 (1994).

25. Zarrinpar, A. & Callaway, E. M. Functional Local Input to Layer 5 Pyramidal Neurons in the Rat Visual Cortex. *Cereb. Cortex* **26**, 991–1003 (2016).

26. Delevich, K., Jaaro-Peled, H., Penzo, M., Sawa, A. & Li, B. Parvalbumin interneuron dysfunction in a thalamo-prefrontal cortical circuit in Disc1 locus impairment mice. *eneuro* ENEURO.0496-19.2020 (2020). doi:10.1523/ENEURO.0496-19.2020
